# Gdaphen, R pipeline to identify the most important qualitative and quantitative predictor variables from phenotypic data

**DOI:** 10.1186/s12859-022-05111-0

**Published:** 2023-01-26

**Authors:** Maria del Mar Muñiz Moreno, Claire Gavériaux-Ruff, Yann Herault

**Affiliations:** 1 Université de Strasbourg, CNRS UMR7104, INSERM U1258, Institut de Génétique, Biologie Moléculaire Et Cellulaire (IGBMC), 1 Rue Laurent Fries, 67404 Illkirch Graffenstaden, France; 2 Université de Strasbourg, CNRS, INSERM, CELPHEDIA, PHENOMIN-Institut Clinique de La Souris (ICS), 1 Rue Laurent Fries, 67404 Illkirch Graffenstaden, France; 3 Present Address: John P. Hussman Institute for Human Genomics, University of Miami, Miller School of Medicine, Miami, FL 33136 USA

**Keywords:** R package, Phenotypic data, Clinical data, Discrimination, Generalized linear models, Random forest, Imputation, Model, Prediction, Machine learning, Bootstrapping

## Abstract

**Background:**

In individuals or animals suffering from genetic or acquired diseases, it is important to identify which clinical or phenotypic variables can be used to discriminate between disease and non-disease states, the response to treatments or sexual dimorphism. However, the data often suffers from low number of samples, high number of variables or unbalanced experimental designs. Moreover, several parameters can be recorded in the same test. Thus, correlations should be assessed, and a more complex statistical framework is necessary for the analysis. Packages already exist that provide analysis tools, but they are not found together, rendering the decision method and implementation difficult for non-statisticians.

**Result:**

We present Gdaphen, a fast joint-pipeline allowing the identification of most important qualitative and quantitative predictor variables to discriminate between genotypes, treatments, or sex. Gdaphen takes as input behavioral/clinical data and uses a Multiple Factor Analysis (MFA) to deal with groups of variables recorded from the same individuals or anonymize genotype-based recordings. Gdaphen uses as optimized input the non-correlated variables with 30% correlation or higher on the MFA-Principal Component Analysis (PCA), increasing the discriminative power and the classifier’s predictive model efficiency. Gdaphen can determine the strongest variables that predict gene dosage effects thanks to the General Linear Model (GLM)-based classifiers or determine the most discriminative not linear distributed variables thanks to Random Forest (RF) implementation. Moreover, Gdaphen provides the efficacy of each classifier and several visualization options to fully understand and support the results as easily readable plots ready to be included in publications. We demonstrate Gdaphen capabilities on several datasets and provide easily followable vignettes.

**Conclusions:**

Gdaphen makes the analysis of phenotypic data much easier for medical or preclinical behavioral researchers, providing an integrated framework to perform: (1) pre-processing steps as data imputation or anonymization; (2) a full statistical assessment to identify which variables are the most important discriminators; and (3) state of the art visualizations ready for publication to support the conclusions of the analyses. Gdaphen is open-source and freely available at https://github.com/munizmom/gdaphen, together with vignettes, documentation for the functions and examples to guide you in each own implementation.

**Supplementary Information:**

The online version contains supplementary material available at 10.1186/s12859-022-05111-0.

## Background

In individuals or animal models suffering from a disease or with another condition, the identification of selective discriminating variables is important to understand the differences between disease and non-disease states, to detect the response to treatments or to discern sexual dimorphism. The current behavioral data are generally complex multifactorial datasets. Those datasets may contain plenty of both qualitative and quantitative information, in some cases structured in groups when several features are measured in the same test. The data recorded can include the performance of each individual in the different tests, or the performance of paired or grouped animals in sociability or mating studies, and can contain extensive medical information about phenotypes, weight, health problems and other molecular records such as gene expression.

The analysis of these data allows to get new insights into the genotype–phenotype relationships and identify key behaviors or clinical biomarkers that are more correlated with genotype, disease state or treatment effect. However, the recorded experimental data have specific particularities and often suffers from several problems making the statistical analysis really challenging.

First, small sample size or number of experimental observations increase the error margins and decrease the confidence in the results and the statistical power over the features [1, 2]. Second, a high number of independent variables referred normally as predictor variables or features, together with the small sample size bring the curse of dimensionality when requiring statistical significance to obtain reliable results and is a challenge due to the high number of features [3]. Thus, there is an increase in the dimensions of the analysis and the available data becomes sparse.

Third, a critical point arises when analyzing data produced by unbalanced experimental designs [4, 5] where for example, the number of observations per group or condition is not balanced or even more, when some variables could not be recorded for all the individuals per group, so there are missing values (often noted as Not available, “NAs”) or when each feature was measured in different individuals that just have in common being members of the same group [6].

Fourth, several features can be recorded in the same tests and some within-test features are highly correlated, thus the not-independent features should be identified and removed as part of the feature selection, to not run into a multicollinearity problem [7, 8]. This is because highly correlated features do not provide new information and instead will add noise that will weaken the model, and even more when considering low samples sizes. In fact, the coefficient estimates of the features will impact on the dependent variable and will give larger standard errors (increasing the type II error) and reduce the efficiency of the model predictions when applied to a new set of data. In addition, within-test features could belong to different distribution families. Then, it should be considered to apply the appropriate statistical analyses to reach accurate results.

Regarding all these core challenges embedded in the experimental data itself, it is clear that a more complex statistical framework is necessary to identify the most discriminative variables among dependent variables and give them reliable and comparable coefficients of importance.

Currently, there is an array of packages in R programming environment that provide tools and functions targeting different parts of the analysis from data cleaning with NAs imputation (MICE [9], amelia [10], missForest [11], Hmisc [12], bcv [13]), correlation analyses (Hmisc [12], corrplot [14], polycor [15], caret [16]), feature selection (MASS [17], caret [16], mlr [18]), statistical modellization (caret [16], glmNet [19], randomForest [20]) and multifactor analyses (PCAmixdata [21], FactoMineR [22], factoextra [23]). However, they are not found together in a joint-pipeline. Thus, for the biologist and medical community that are non-statisticians with little knowledge about machine learning techniques and functions, it can be difficult to decide and implement these methods. Moreover, in most cases the visualizations produced by existing tools cannot be used in publications as they are difficult to read, explain or customize in both the colors and sizes of the plots.

To address these limitations and answer to the community needs, we developed Gdaphen (Genotype discrimination using phenotypic features), a pipeline in R containing a suite of functions capable of dealing with all the key analytical steps. Gdaphen includes, from preprocessing steps such as data anonymization or imputation of missing values, to feature selection where a new modeling method is implemented based on selecting features contributing more than 30% to explain the variance in the data, and visualization. Gdaphen is freely available on GitHub (https://github.com/munizmom/gdaphen; https://github.com/YaH44/GDAPHEN).

Gdaphen was especially designed to be used by the communities working with data collected from (1) genetic models carrying single gene mutations, (2) models with multiple gene modifications like duplications and/or deletions, (3) models carrying a gene dosage mimicking the one observed in diseases like Down syndrome [24]. Indeed, Gdaphen includes modelling methods based first on GLMs, to elucidate which variables are discriminative of dosage effects as the method will identify linear relationships between the dependent and explanatory variables. In addition, GLMs allows to model data from the exponential family not limiting all the recorded variables to follow strictly a Gaussian distribution. Second, to identify the best predictor variables even if there is a non-linear relationship between the dependent and explanatory variables, we implemented a machine learning bootstrapping-based algorithm, the random forest classifier.

Next, to overcome the curse of dimensionality in the data imputation, Gdaphen uses another machine learning bootstrapping-based algorithm aregImpute. Gdaphen allows to perform the discrimination analysis using three different models of features. First, Gdaphen considers all the features in a dataset and perform two methods of features selection (1) eliminating the highly correlated variables (2) selecting the variables accounts for more than a certain percentage of the variability in the dataset. Thanks to these multimodal analyses, Gdaphen can provide an in-depth analysis of all the features, focus on the identification of multicollinearity to decipher its biological relevance and assess the necessity, efficiency, and suitability of employing feature selection methods considering the data at hand.

Finally, in the visualization module, Gdaphen allows to resize or choose the colors assigned to each feature or group of features for each model analyzed. The plots produced can be used directly in the publications with good quality and readability. Moreover, as the color representing thoses variables can be chosen, the comparison of results between your different publications and analyses is made easier.

Overall, Gdaphen main goal is to provide the guided computational framework to answer the following questions.

1. Assess the efficiency reached by each classifier (GLM or RF), on the three different input datasets, the original dataset carrying highly correlated features and the datasets ready for the analysis generated by performing the two feature selection methods. The feature selection methods help to denoise the data and reach statistically reliable results.
2. Assess feature correlation and provide the statistical assessment for each found correlated pair.
3. Identify the grouped/non-grouped features that are more relevant to discriminate between genotypes, sexes, mouse line backgrounds or treatments effects. And in the case of gene or treatment dosages, identify the features following a linear dosage effect.

**Fig. 1:**
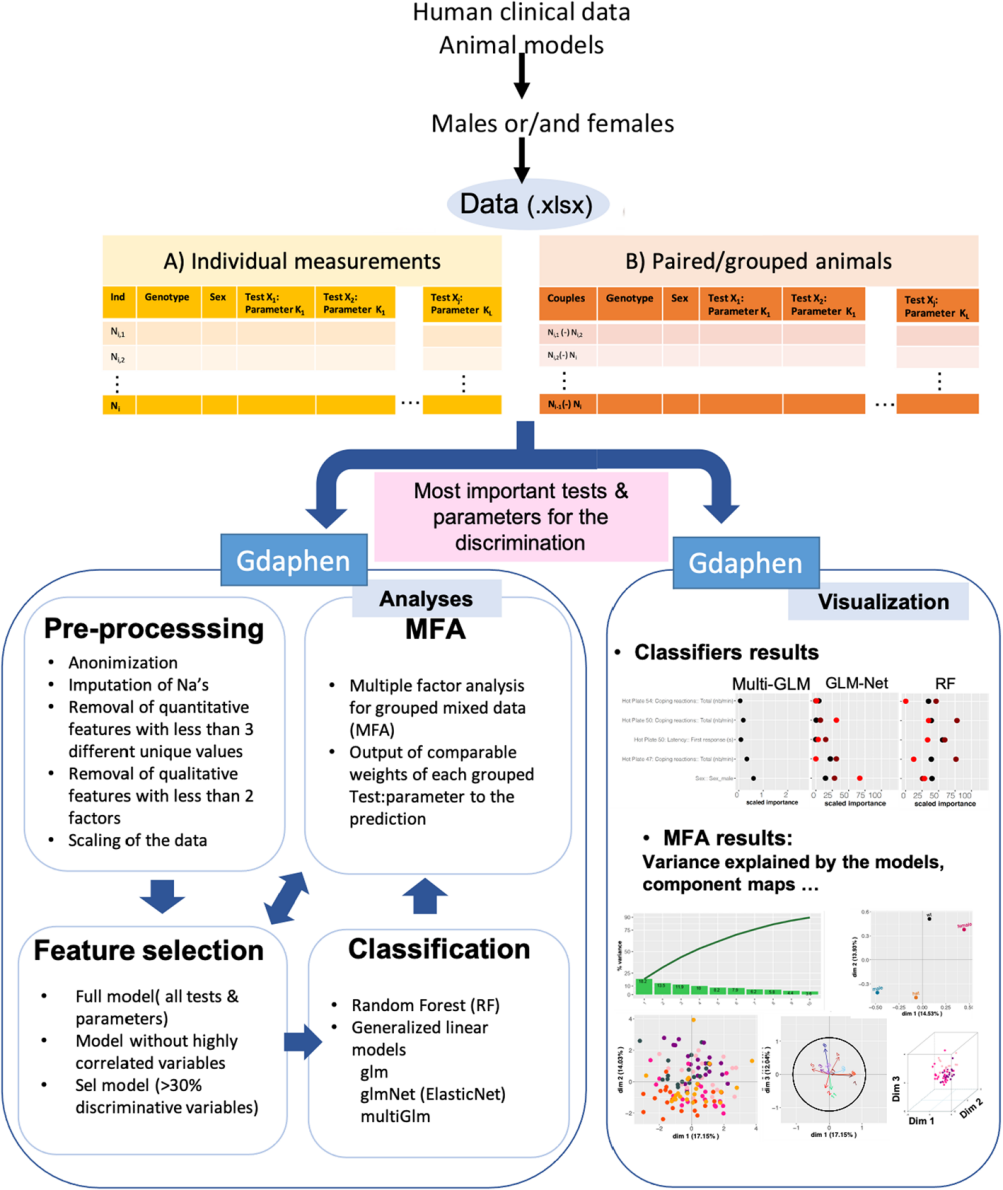
Gdaphen workflow. Scheme highlighting the functionalities that Gdaphen can help to implement. From the data organized with individuals in rows and parameters measured in columns, Gdaphen comprises three modules. The first module deals with data pre-processing to shape the data in the input needed to perform the different analyses. Next, the module Analysis is where MFA, feature selection and classification strategies are performed. Last, the module visualization contains the functions that will generate the plots ready for the publications. As shown in the figure, a dotplot is implemented to show the classifiers results in the importance of each variable to the discrimination of your variable of interest. Then the MFA results are shown using different visualizations

In this paper we give an overview of Gdaphen main features (Fig. 1; Table 1) and show two examples of the application of Gdaphen to phenotypic datasets of mouse models of neuropathic pain. The datasets show increased complexity and some results of the application of GDAPHEN are published as part of the deep statistical analyses performed over the phenotypic characterization of these models [25, 26].

**Table 1:** Gdaphen goals and more relevant features described for each module: pre-processing, analysis and visualization

Gdaphen module	Step	Goal	Relevant features
Pre-processing	Pre-processing	Prepare the data for the analysis	Anonymization of the samples/ individuals in case we work with human datasets. Renaming the identifiers, noted as "Ind" of each animal if there are duplicated when considered per genotype/sex Imputation of NAs using two methods (a) the mean if only one value is mising. (b) Using aregImpute from Hmisc package [12] to perform a Multiple Imputation using Additive Regression, Bootstrapping, and Predictive Mean Matching Removal of quantitative features with less than 3 unique values Removal of qualitative features with less than two factors Scaling of the data to standardize the features
Analysis	Feature selection	Analyze the correlation in the explanatory variables or features	Identification of the highly correlated variable setting up a threshold of correlation decided by the experimenter/analyst
		Apply methods for pre-selection of features to decrease the noise of the data using the low correlated features	Methods for pre-selection of features based on: (a) The removal of highly correlated features (b) A novel approach selection of explanatory features contributing to the discrimination more than a 30% after running the MFA analysis
Analysis	Multi factor analysis (MFA)	A multiple factor analysis (MFA) to identify the weight of each feature/group of features to the prediction	Perform a Multivariate Analysis of Mixed Data using the package PCAmixdata [21] the function MFAmix. Some of the outputs of the function are the following: Squared loadings Eig matrix with eigenvalues Results for qualitative or quantitative features apart Results for the levels of each qualitative feature Coordinates of groups Partial individual coordinates The coefficients of the linear combinations of features
		Cosine similarity distances to identify the degree of similarity between the dependent feature and the explanatory features in the PCA dimensional space. Method developed by Escoffier and Pages [28]	Calculate the cosine similarity distance matrixes
Analysis	Classification	Several classifiers’ algorithms to identify the main discriminative explanatory variables some specially elected to identify variables affected by gene dosage as GLM, multiGLM and GLMNet. And a supervised method Random Forest	Generalized linear regression model, noted as GLM. Computed by the function train of the package caret, setting the parameter method = "glm” Penalized Multinomial Regression also called multinomial log-linear models via neural networks method, noted as multiGLM using the function multinom from the package net [27] Elastic Net method, noted as GLM-Net model, fits a generalized linear model in more than two factors by using a penalization maximum likelihood by combination of the ridge and lasso shrinkages and optimizing the parameter lambda and alfa. Is computed using the function train of the package caret, setting the parameter method = "glmnet”. Requires de package glint and Matrix. The theory is described in Friedman et al. [29], Simon et al. [30] A random forest, noted RF, supervised algorithm. Is computed using the function train of the package caret, setting the parameter method = “rf”
Analysis			Calculi of the mean, SD, error and upper and lower confidence intervals for each feature. Computed using the function ggparcoord from the ggally package [31]
Visualization	Classification results	Asses the efficiency of the classifier We provide a table and plots where the scaled importance of each feature relativized to the most important one is shown for all the variables	Produce plots and tables containing the scaled importance of each feature after running the glm/glmNet/multiGLM/RF classifiers for the three-input data (features selected and not selected)
Visualization	MFA results	9 plots that show the individual observations distribution in the in the top dimensions of the three main principal components of the PCA (quantitative data)/MCA (qualitative data) and the contribution of each feature or grouped feature in the main principal components	1.Variance explained by top ten PCA components & cumulative variance 2.Variables contribution/correlation with the three main principal components: grouped-features correlation partial axes plot 3.Grouped features correlation to the three main principal components dimensions PCA plot 4.Observations distribution to the classifier discrimination of qualitative variables in the principal components three top dimensions using 2D and 3D PCA plot visualizations 5.Squared loadings of the quantitative and qualitative features and the principal components 6.Features correlation with the three main principal components: all variables’ correlations 7.Qualitative features discrimination: Qualitative features distribution in the main dimensional spaces 8.Parallel coordinates plots with scaling and nots scaling of features to visualize the differences between your dependent variable factors 9.Scaled importance of each variable after running the glm/glmNet/multiGLM/RF classifiers

## Results

We now describe in detail Gdaphen framework main features separated in three modules, (1) the pre-processing, (2) the analysis and (3) the visualization modules.

### Pre-processing module

#### Data import and preprocessing

The first and more important step is to collect all the phenotypic/clinical features or gene expression measurements variables recorded for each individual and prepare the data for the analysis. As the scientific community finds more suitable to work and manipulate excel files, Gdaphen takes as input data an excel table (file extension “.xlsx”). The file should contain the collected information per individual in rows and all the features or variables recorded in columns. Considering that in some behavioral or clinical tests several variables were recorded in the same tests, we can group those variables with the same “group label” to be able to identify the importance for the discrimination of (1) each variable alone, (2) the overall contribution of the group. This grouping is decided by the analyst and specified in the excel file by using the “::” separator. After the importing step, the input file is stored in a dataframe object that will be subjected to several pre-processing steps to make it complete and ready for running Gdaphen. Gdaphen will help you perform the following tasks:

- *Identifier anonymization* Gdaphen will automatically perform this task. The experimenter can choose to perform a data identifier anonymization, creating a unique identifier per subject or instead use the identifiers provided in the input file if they have not duplicated values. In this regard, Gdaphen will consider two individuals have a duplicated identifier, noted as “IND” after regarding the features selected as conditionate like genotype, sex and treatment state.
- *Imputation of missing values if they exist* There are several methods of imputation of missing values (NAs) widely used in biology. The most common one when only one NA exist, is to impute using the mean or the median for each group of individuals. The group is shaped considering the features selected as conditioning, (like genotype, sex and treatment state). In this case the experimenter can already replace the missing value by his chosen mean/median value. Although we recommend that no imputation method should be used if the number of individuals per group is less than 10 or if the distribution of the values is broad and the standard deviation too high. On these cases, if imputation is really insisted on, it could be better to select a random number considering your sampling size.
  Instead, if more than one missing value exist considering the features selected as conditioning, Gdaphen guides you in the examples to implement a method for imputation using Additive Regression, Bootstrapping, and Predictive Mean Matching based on closest random sampling implemented over the aregImpute function from the Hmisc R package [12]. One of the interesting features that made us select this method of imputation is that it won’t select the mean or closest to mean values for the group, instead it computes the K number of closer neighbors and randomly chosen, and as many as you need to impute. Introducing this randomness in the selection, allowed us to introduce a model closer to the variability observed on biological replicates. Finally, in this module Gdaphen will automatically perform the tasks below:
- *Consider quantitative variables with enough different unique values* to calculate minimal errors or standard deviation.
- Consider qualitative variables with at least two different categories to perform the discriminative analysis.
- *Standardization of the data* by scaling: this step is necessary as each independent variable has a different range of values that are observed. The re-scaling allows to calculate the contribution of each variable in a comparable way as all variables will have the same range of values observed and/or the same variance.

### Analysis module

#### Data classification and identification of the most relevant variables to the discrimination

The classification of the data in one of the possible set of “classes” or categories of a dependent variable previously defined by the analyst is the aim of using the classifiers. We decided to use two different classifiers to give answer to two different questions.

A. A generalized linear model or Lasso and Elastic-Net Regularized Generalized Linear Model, noted as GLM or GLM-Net. Generalized linear models are more suitable to discover variables influenced by gene dosage effects as they will identify which phenotypic variables or “predicting variables”. In addition, GLMs can discriminate since their linear combination is influencing the value of the response variable. Moreover, the use of these models instead of just linear models allow to consider variables following not only a normal distribution but a distribution from the exponential family. This broadens the scope of the features that can be recognized as relevant to the prediction even when the sample size is low.
B. A random forest, noted RF, is a supervised algorithm that will recognize the most relevant phenotypic variables for the discrimination even though those features may not follow a distribution from the exponential family and even if non-linear relationships exist between the predicting variables and the response variable.

Both functions are taken from the caret and nnet R packages [16, 27]. As an output a table is obtained with all the predictive variables measures of importance in a relative scale to the most important one that have a maximum value of 100. In addition, this analysis provides an easy visualization in a dot plot shape, where it is straightforward to identify the top discriminant variables for each factor level of the dependent variable.

#### Identification of grouped features contributions to the predictions

We included a Multiple Factor Analysis (MFA) to assess the groups or “test-specific” contribution to the discrimination and the weight of each individual/grouped variable to the prediction. This method can deal with groups of variables, both qualitative and quantitative, recorded from the same individuals. The MFA performs a normalization or “weighting” on each group by dividing all the variables belonging to the group by the first eigenvalue coming from the principal component analysis (PCA) of the group. This procedure is done to avoid giving more weight to groups that have recorded a higher number of parameters. Then a PCA on all the weighted variables is applied to (1) identify the correlation between the qualitative or quantitative variables grouped or ungrouped, and the principal component dimensions, (2) identify the individual coordinates of each observation on the PCA dimensions. The method is implemented using the MFAmix function from the PCAmixdata R package [21]. Moreover, we chose a vectorization visualization approach as the one implemented in PCAmixdata were we included the cosine similarity distance to further highlight the parameters that follows the same or opposing trajectory for each category of our dependent variable. Thus, those parameters are contributing to the separation of the individual data on the same dimensions defined by their cosine similarity distance.

#### Pre-selection of phenotypic variables

Gdaphen was designed to perform the analyses using all the features provided by the experimenter. We noted the results of this analysis as the “full model”. In addition, Gdaphen implements two methods to pre-select the explanatory variables considered.

1. The first method runs by default and is advised to use always to identify and remove the highly correlated variables as they lack independency and should not be included in any statistical assessment. The highly correlated variables could be regarded in a table where the correlation matrix is provided highlighting those correlated with a higher index than the one set off by the analyst as a threshold. Identifying correlated variables and further understanding why the correlation exists from a biological point of view can be extremely important to understand the in-depth nature of the variables recorded. In addition, can aid in identifying interesting and biologically meaningful relationships in the data. For example, in mice weight and sex are two variables that are highly correlated. We recommend for downstream classification analyses to not allow variables with a correlation index higher than r = 0.75.
2. The second pre-selection method is an optional one, the main purpose is to reduce the p > n dimensionality problem by reducing the number of explanatory variables (p) compared to the number of observations (n) and increase the data variance explained by the models built by the classifiers to finally strengthen the classification. This pre-selection is done based on the phenotypic variables identified to contribute to the discrimination at more than 30% after running the MFA analysis using all variables without the highly correlated ones and measuring the correlation between the quantitative ungrouped phenotypic variables with the main three dimensions of the PCA. To assure that Gdaphen is performing as good with this model than with the model created using all the variables, we calculated the variance of the data we are able to explain over the first 10 dimensions and the accuracy of the models to answer to how well they can correctly predict each individual observation to the correct class of the dependent variable. The results are visually highlighted in the plot labelled cumulative variance

### Visualization module

Resulting from the analysis, Gdaphen generates nine plots that are ready to be included in formal publications and where we leave certain aesthetic parameters to be defined by the analyst to assure the good sizing of their plots depending on the number of explanatory variables and number of categories of dependent variables.

1. Grouped features correlation to the three main principal components dimensions partial axes plot. Stored in partialAxes_Plot1 folder
2. Variables contribution/correlation with the three main principal components: grouped-features correlation PCA plot. Stored in groupContribution_Plot2 folder.
3. Observations distribution to the classifier discrimination of qualitative variables in the principal components three top dimensions using 2D and 3D PCA plot visualizations. Stored in individualCoordinates_Plot3 folder
4. Squared loadings of the quantitative & qualitative features and the principal components. Stored in sqload_Plot4 folder.
5. Features correlation with the three main principal components: all variables’ correlations. Stored in quantitativeVarCoordinates_Plot5 folder.
6. Qualitative features discrimination: Qualitative features distribution in the main dimensional spaces. Stored in levelsComponents_Plot6 folder
7. Parallel coordinates plots with scaling and nots scaling of features to visualize the differences between your dependent variable factors. Stored inside the paralelPlot folder.
8. Scaled importance of each variable after running the glm/glmNet/multiGLM/RF classifiers. Stored inside the importance_variables folder.
9. Variance explained by top ten PCA components & cumulative variance. Stored in cumulative Variance folder.

#### Benchmarking Gdaphen: results

We have run Gdaphen pipeline on the phenotypic data scored for two novel mouse genetic models showing an increased pain behavior (1) *Scn10a*^*G1662S*^ Point Mutation, and (2) *Scn9a*^*R185H*^ point mutation. We also tested successfully Gdaphen with RT-qPCR data (data not shown, the sample size was low).

A part of the analysis performed on the mouse models for *Scn10a* [25] and *Scn9a* [26] genes is already available in the publications containing the molecular and phenotypic characterization of these models.

**Fig. 2:**
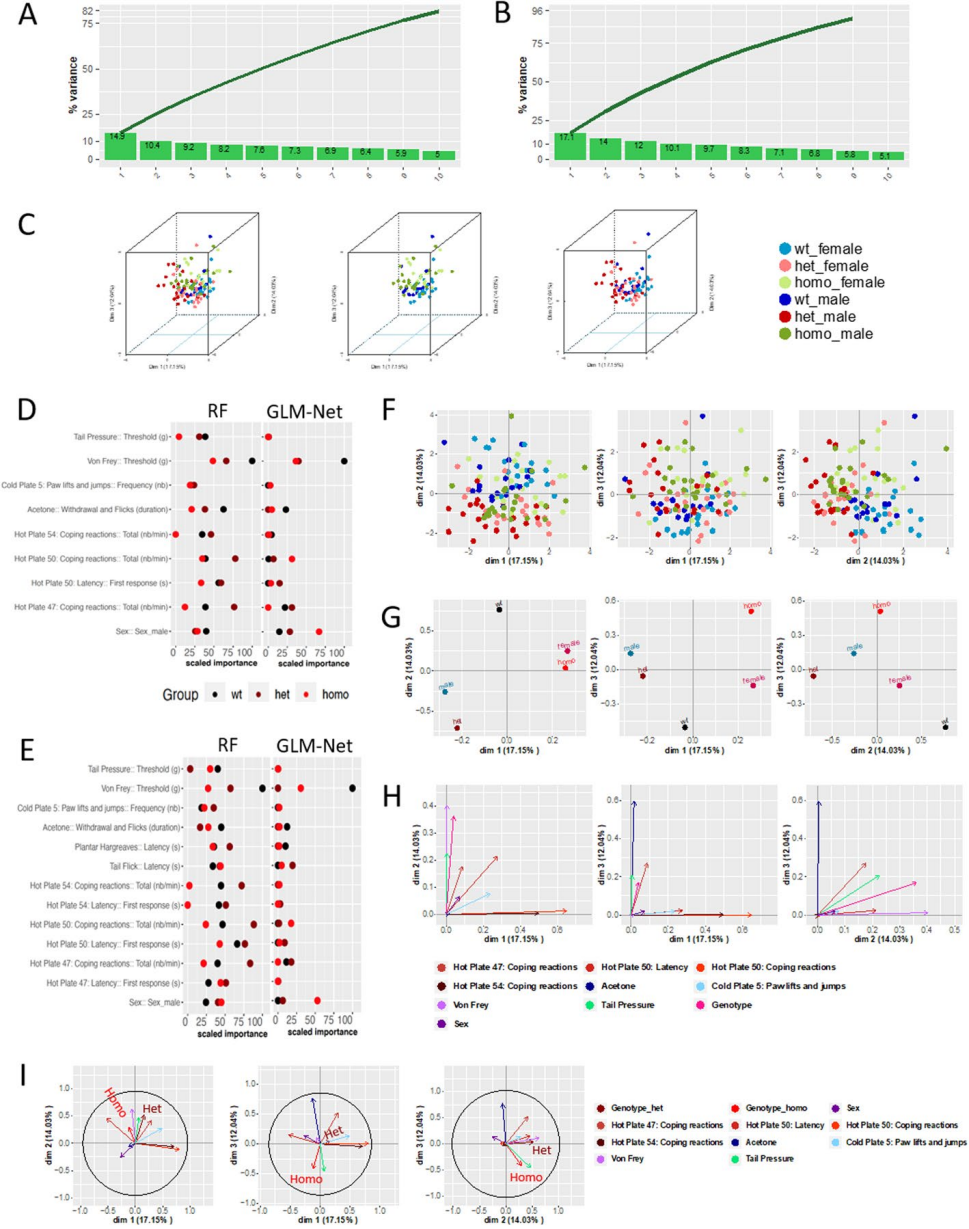
Example *Scn10a*^*G1662S*^ Gdaphen analysis. **A** Variance explained by the top ten PCA components and cumulative variance using the full model containing the 14 variables. **B** Variance explained by the top ten PCA components and cumulative variance using the sel30% model containing 9 variables. **C** 3D-PCA plots showing the individuals clustering on the first 3 dimensions coloring based on each genotype and sex combination. Left panel shows all the genotypes and sexes, the middle plot shows only wild type and heterozygous data, and the right plot shows only wildtype and heterozygous data. **D** Classifiers results for the 9 variables included in the Sel30% model showing the most important variables to genotype discrimination after scaling to the top discriminative one. **E** Classifiers results for all the 14 variables included in the full model showing the most important variables to genotype discrimination after scaling to the top discriminative one. **F** 2D-PCA plots showing the individuals clustering on the first 3 dimensions and colored based on each genotype and sex combination. **G** Categorical variables discrimination component map. The panels show the distribution in 2 dimensions of the categorical variables PCA coordinates calculated in the MFA analysis using the MFAmix function from PCAmixdata R package [21]. **H** Square loadings plot with coordinates calculated in the MFA analysis using the MFAmix function from PCAmixdata R package [21]. **I** Cosine similarity distance coordinates drawn in each principal component for the selected 30% variables of the analyses calculated by the MFAmix function. The arrow length measures the contribution of each variable to the discrimination on each dimension. Arrows that follow similar trajectories (stronger cosine similarity distance) contribute to the discrimination of the data in the same dimensions

**Fig. 3:**
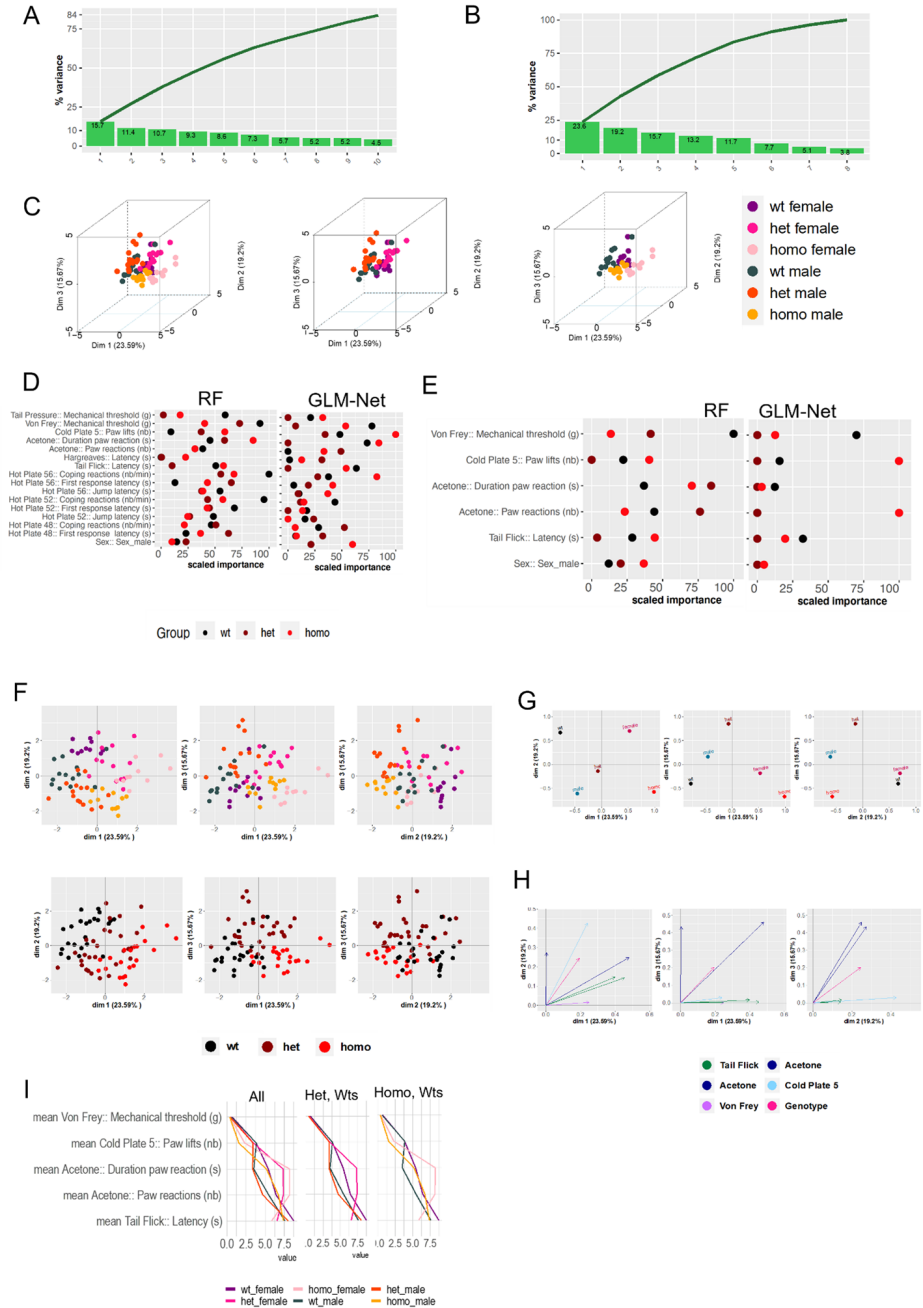
Example *Scn9a*^*R185H*^ Gdaphen analysis. **A** Variance explained by top the ten PCA components and cumulative variance using the full model containing the 14 variables. **B** Variance explained by top the ten PCA components and cumulative variance using the sel30% model containing 9 variables. **C** 3D-PCA plots showing the individuals clustering on the first 3 dimensions and colored based on each genotype and sex combination. Left panel shows all the genotypes and sexes, the middle plot shows only wild-type and heterozygous data, and the right plot shows only wild-type and homozygous data. **D** Classifiers results for the 6 variables included in the Sel30% model showing the most important variables to genotype discrimination after scaling to the top discriminative one. **E** Classifiers results for all the 18 variables included in the full model showing the most important variables to genotype discrimination after scaling to the top discriminative one. **F** 2D-PCA plots showing the individuals clustering on the first 3 dimensions. On the upper panel coloring is based on genotype and sex combinations. On the lower panel coloring is just by genotype. **G** Categorical variables discrimination component map. The panels show the distribution in 2 dimensions of the categorical variables PCA coordinates calculated in the MFA analysis using the MFAmix function from PCAmixdata R package [21]. **H** Square loadings plot with coordinates calculated in the MFA analysis using the MFAmix function from PCAmixdata R package [21]. **I** Parallel plot showing the non-scaled results of the most influencing variables to the discrimination colored by genotype and sex showing the mean of the variable per group of genotype and sex. In the left parallel, the plot using all the genotypes and sex data. The middle panel shows only heterozygous and wild-types and the right panel only homozygous and wild-types

1. For the *Scn10a*^*G1662S*^ murine model, 112 animals including males and females (57 females and 55 males) and a total of 36 wild-type, 39 heterozygous, 37 homozygous animals were challenged in a phenotypic pipeline and the data from 14 variables was collected for the analysis. These variables included genotype, sex, and 12 phenotypic traits (Additional file 2: Table S2). None were correlated more than a 75% (Additional file 3: Table S3) and so we considered there was no strong multicollinearity and proceeded to perform the second feature selection. Seven variables plus sex and genotype were contributing to explain the variability of the dataset in more than a 30% and were thus identified as the most discriminative variables for the three genotypes. When examining the cumulative variance considering the first 10 dimensions (plot cumulative variance, Fig. 2A, B) we observed that feature selection helps to denoise the data. Indeed, the sel30% model shown in Fig. 2B explained a higher % of the variance of the data (100%) than using the full model containing 14 variables (86%) shown in Fig. 2A. The individual clustering based on genotype and sex, shown more differences between homozygous and wildtypes in the 3D-PCA space and a slight deviation of the center of the clusters when considering female observations or male observations (Fig. 2C). Indeed, the first two dimensions appear to explain the most the genotype and sex variability (Fig. 2F, G), where dimension 1 helps more in discriminating the homozygous and dimension 2 and 3 the three genotypes. In fact, it can be clearly seen at the spread clustering that the mutants (homozygous and heterozygous) show differences in comparison with the wild types without producing a strong phenotype difference (Fig. 2C, F). Looking at the results for classifiers, nine variables (sel30% model) were selected: acetone test, coping reactions and latency on 50 °C hot plate, coping reactions on the 54 °C and 47 °C hot plate, number of paw lifts and jumps on a 5 °C cold plate, sex, tail pressure and von Frey (Fig. 2D). The classifiers GLM-Net and RF identified von Frey as the main variable for genotype discrimination. In addition, both classifiers also identified acetone test, number of coping reactions and latency time on 50 °C hot plate as key explanatory variables. Tail pressure was only identified by RF. When compared to the full model, the variables highlighted in the sel30 model are emphasized. In addition, tail flick and plantar Hargreaves can help in the discrimination of 2 out of the three genotypes. However, these two variables do not produce a better discrimination analysis considering the variance explained by the models. In support of the classifiers results for the sel30% model, the multi factor analysis (MFA) square loading plot (Fig. 2H) show that von Frey and number of copying reactions in Hot plate 47 and 50 degrees are the most strongly influencing variables in our dataset in dimensions 1 and 2 and dimensions 2 and 3. Tail pressure was also contributing weakly to those dimensions for the genotype discrimination. In addition, the number of paw-lifts and jumps on the 5 °C cold plate contributes to the discrimination in dimensions 2 and 3. Coping reactions on the 50 °C and 54 °C hot plates contributed the most in dimension 1 (Fig. 2H). Moreover, examining the correlation between the location of each vectorized variable with each genotype on the principal components by the cosine similarity distance (Fig. 2I), we reach the same conclusions.
2. For *Scn9a*^*R185H*^ murine model, 73 animals including males and females (34 females and 39 males) and 24 wild-type, 28 heterozygous and 21 homozygous individuals, were challenged in a specific phenotypic pipeline and the data from 18 variables was collected for the analysis. Those variables included genotype, sex, and 16 phenotypic traits (Additional file 4: Table S4). None were correlated more than a 75%. Six variables were identified as the most relevant (sel30%) to discriminate the three genotypes. These variables were von Frey, number of paw lifts on cold plate, number of paw reactions and duration of paw reaction in the acetone test, latency in tail flick test and sex (Fig. 3E). When comparing the cumulative variances (Fig. 3A, B) the sel30% model explained a higher % of the variance of the data (100%) than using the full model containing 18 variables (84%).

The individual clustering based on genotype and sex showed more differences between homozygous and wild-types in the 3D-PCA space with heterozygous in between, and the sex differences was mainly collected in dimension 2 (Fig. 3C, F). In fact, the first dimension mainly explains the genotype differences with heterozygous clustering in between the other genotypes correlating well with the milder phenotypes observed in this model. In addition, dimension 3 seems to differentiate well the heterozygous from the other two genotypes (Fig. 3F, G). The classifiers, out of the six variables selected with GLM, did identify the numbers of paw lifts in the cold plate as the most discriminative variable. The other variables important for the discrimination were von Frey, the duration of paw lifts in the acetone test and the tail flicks, all following a linear gene dosage effect. In addition to these, RF also selected the number of paw reactions in the acetone test. Moreover, considering the full model, most of the variables scored in the hot plate could contribute to discriminate between certain pairwise combinations of genotypes. However, considering that those variables did not produce a better discrimination as shown by the variance explained by the models, they were not included in the sel30% model (Fig. 3A, B). In support of the classifiers results for the sel30% model, the multi factor analysis (MFA) square loading plot (Fig. 3H) shows that numbers of paw lifts in the cold plate contribute the most in dimensions 1 and 2. The two parameters scored in the acetone test contribute the most and highly similarly in dimension 2 and 3 and just the duration of paw lifts in dimension 1 too. Von Frey contributed only to dimension 1 and tail flick in dimension 1 and 2. Finally, we show also the visual aid that the parallel plot can provide to identify the differences between the scored variables and the genotypes-sex, before or after scaling you can visually discriminate the differences pointing to the same results already discussed above. In addition, we can visually discriminate easily using the parallel plots the most discriminative variables for each condition of interest (Fig. 3I).

## Conclusions/discussion

Taking advantage of the R programming software and the existence of state-of-the-art packages to implement statistical analyses, Gdaphen provides an open-source integrated computational framework for variable discrimination based on phenotypic data that is easily accessible for medical and behavioral researchers. Considering the real challenge and burden the analysis of these data represents for our community, we saw the need to create and distribute this package. Hence Gdaphen, definitively a versatile and adaptable pipeline can become a valuable implementation in the R phenotypic-based statistical research ecosystem.

Moreover, Gdaphen gathers functions for the analysis and visualization of the most important predictor qualitative and quantitative variables for the discrimination between groups. In addition, Gdaphen allows the analysis of grouped variables where the analyst can freely establish the groups. Finally, Gdaphen includes examples to guide you along the pipeline to allow non expert R users to perform their own analyses and take their own decisions depending on their datasets.

A certain limitation of this pipeline exists in the fact that we did not introduce phenotypic regression algorithms to predict the expected phenotypes based on the associations between single nucleotide variants (SNVs) identified by genome-wide association studies (GWAS) data, splicing or expression quantitative trait loci (QTLs) and linkage disequilibrium to mention some. Instead, we decided to implement uniquely classification analyses, because our final goal was to identify categorial responses in the dependent variable as for example genotype, sex, treated versus controls, etc. On these cases we use as explanatory variables data measured from phenotypic analyses, clinical data, histopathological measurements, or genetic information in shape of gene expression measurements coming from RT-qPCR, dd-PCRs or RNA-Seq analyses. Thus, Gdaphen is not suitable in the package current form to address phenotypic regression problems although this can be part of a future implementation.

The relevance of Gdaphen implementation and suitability to provide better insights into the data is without question, as proven in the two example cases shown here. The biological relevance can be even higher if dealing with analyses of multiple diseases or more complex experimental designs containing several variables of interest to study like genotype, sex, different diseases models or treatments versus control cases. Gdaphen can aid in the decision of which variables to score and preserve or disregard in phenotypic pipelines. This will allow to avoid losing experimental time in scoring variables or test that would not have a strong impact in the discrimination. Thus, there is a lot of potential in applying Gdaphen for complex datasets to unravel the biology under them and to establish experiment pipelines.

## Methods

### Implementation

Gdaphen is a R pipeline that allows the identification of the most important predictor qualitative and quantitative variables for a specific dependent variable discrimination in animal models of diseases. The dependent variable can be freely setup by the analyst but should be of categorical nature. As an example, Gdaphen was primary developed to identify the variables most important for genotype discrimination or to uncover the variables more influenced by sex or treatments responses. Gdaphen can be run in RStudio or in shell R. Gdaphen pipeline is freely available on GitHub accompanied by one vignette in classical format and another as an RStudio project object (https://github.com/munizmom/gdaphen; https://github.com/YaH44/GDAPHEN).

Gdaphen main features include the availability in the same pipeline of the functionalities and visualizations as summarized in Fig. 1 and Table1. The full list of packages and dependencies needed to run Gdaphen is shown in Additional file 1: Table S1.

### Availability and requirements

Project name: Gdaphen: R pipeline to identify the most important predictor qualitative and quantitative variables from phenotypic data for the discrimination of your variable of interest. Project home page: https://github.com/munizmom/gdaphen. https://github.com/YaH44/GDAPHEN. Operating system: Mac/Linux/Windows. Programming language: R. Other requirements: Installation of R runtime library R2019a (9.6) and several R packages as specified in Additional file 1: Table S1. Can run in bash R or R Studio. License: GNU GPL 3.0. Any restrictions to use by non-academics: None.

## Supplementary Information

**Additional file 1. Table S1: **List of R packages implemented on Gdaphen.

**Additional file 2. Table S2: **Input data of *Scn10aG1662* mutant mouse line. Each row shows data from one individual and each column all the features quantified and metadata.

**Additional file 3. Table S3: **Correlation matrix results for all the variables in the *Scn10aG1662* mutant mouse line. The values shown are r values.

**Additional file 4. Table S4: **Input data of *Scn9aR185H* mutant mouse line. Each row shows data from one individual and each column all the features quantified and metadata.

## Data Availability

Data and source code in an R package are available at https://github.com/munizmom/gdaphenhttps://github.com/YaH44/GDAPHEN

## Abbreviations

2D: Two dimensions
3D: Three dimensions
NA/NAs: Missing values
IND: Individuals or independent observations
DD-PCR: Digital droplet polymerase chain reaction
GLM: Generalized linear model.
GLM-Net: Lasso and Elastic-Net Regularized Generalized Linear Models
GWAS: Genome-wide association studies
MFA: Multi factor analysis
Parameters: Are the variables scored in the phenotypic test or the genes measured in the gene expression experiments
PCA: Principal component analysis
QTLs: Quantitative trait loci
R: R software for statistical computing
RF: Random forest
RTq-PCR: Real time quantitative polymerase chain reaction
SNVs: Single nucleotide variants
Variables: Are the parameters scored in the phenotypic test or the genes measured in the gene expression experiments
*Scn9a*^*R185H*^: Mouse model with a point mutation identified in chronic pain patients and found to produce a pain phenotype in the mutant mice
*Scn10a*^*G1662S*^: Mouse model with a point mutation identified in chronic pain patients and found to produce a pain phenotype in the mutant mice
